# Artificial intelligence for automated detection of large mammals creates path to upscale drone surveys

**DOI:** 10.1038/s41598-023-28240-9

**Published:** 2023-01-18

**Authors:** Javier Lenzi, Andrew F. Barnas, Abdelrahman A. ElSaid, Travis Desell, Robert F. Rockwell, Susan N. Ellis-Felege

**Affiliations:** 1 Department of Biology, University of North Dakota, Grand Forks, ND 58202 USA; 2 School of Environmental Studies, University of Victoria, Victoria, BC V8W 2Y2 Canada; 3 Department of Computer Science, University of North Carolina Wilmington, Wilmington, NC USA; 4 Department of Software Engineering, Rochester Institute of Technology, Rochester, NY USA; 5 Vertebrate Zoology, American Museum of Natural History, New York, NY 10024 USA

**Keywords:** Computational models, Computational biology and bioinformatics, Ecology, Zoology, Ecology, Engineering

## Abstract

Imagery from drones is becoming common in wildlife research and management, but processing data efficiently remains a challenge. We developed a methodology for training a convolutional neural network model on large-scale mosaic imagery to detect and count caribou (*Rangifer tarandus*), compare model performance with an experienced observer and a group of naïve observers, and discuss the use of aerial imagery and automated methods for large mammal surveys. Combining images taken at 75 m and 120 m above ground level, a faster region-based convolutional neural network (Faster-RCNN) model was trained in using annotated imagery with the labels: “*adult caribou*”, “*calf caribou*”, and “*ghost caribou*” (animals moving between images, producing blurring individuals during the photogrammetry processing). Accuracy, precision, and recall of the model were 80%, 90%, and 88%, respectively. Detections between the model and experienced observer were highly correlated (Pearson: 0.96–0.99, *P* value < 0.05). The model was generally more effective in detecting adults, calves, and ghosts than naïve observers at both altitudes. We also discuss the need to improve consistency of observers’ annotations if manual review will be used to train models accurately. Generalization of automated methods for large mammal detections will be necessary for large-scale studies with diverse platforms, airspace restrictions, and sensor capabilities.

## Introduction

Drones offer a variety of advantages that make them a powerful tool for wildlife ecologists^1,2^. In the past, it has been challenging to obtain data of animal counts spatially and temporally because aircraft missions and satellite images are expensive, and ground-based surveys in many cases are restrictive in terms of accessibility to sites, the areas that could be covered, and the low cost-effectiveness ratio. More recently, drones have emerged as a highly cost-effective tool that allows researchers to reduce survey costs, notably increasing the amount of high-quality information^3–5^. Additionally, drones can offer a non-invasive technique that reduces disturbance in comparison with traditional approaches^6,7^. For these reasons, this technology is being increasingly adopted by wildlife ecologists.

Studies about species detection, abundance, distribution, behavior, and reproduction of terrestrial and marine vertebrates have been growing in the past 2 decades using drone technology^3,4,8,9^. In particular, studies using drones have been carried out in terrestrial mammals, mostly on large herbivores^10–18^. Most of these studies have been conducted in African ecosystems, like the savannas; however, studies on terrestrial mammalian herbivores in the wild are still lacking in Arctic and sub-Arctic ecosystems. These are logistically and financially challenging regions^19^, where the survey area needed to be covered is usually very large, and weather conditions make occupied survey flights dangerous^20^ and satellites unreliable^21^. Fortunately, drones can ameliorate all three of those problems, and could be used for conservation, research, and monitoring in these challenging environments.

One species of conservation interest is caribou (*Rangifer tarandus*) where population declines appear associated with human activities along its distributional range^22^. Available methodologies used to monitor caribou populations such as collaring or monitoring with occupied aircrafts, although useful, could be disruptive to individuals, financially challenging, and certainly logistically intensive^23^. The use of alternative methodologies, such as drones, to study caribou in sub-Arctic habitats of northern North America, has been assessed by Patterson et al.^24^. These authors evaluated the use of drones as a methodology to manually detect and count surrogate caribou in natural habitats. However, to our knowledge drone technology has not been empirically evaluated with wild caribou. Additionally, the generation and management of large amounts of raw data and manual counting from imagery are still time consuming, inefficient, and error-prone, decreasing the benefits of the technology^25,26^. Therefore, efficient and cost-effective approaches to detect and count wild caribou could be advantageous, because its broad distribution requires access to remote locations and to cover extensive (in the range of millions of km^2^) sections of land^27^. This situation imposes a challenge for researchers in their efforts to acquire and rapidly analyze data. As a result, monitoring that incorporates drones also requires the development of automated procedures to provide accurate and timely information for wildlife managers^23^.

One approach to facilitate detection and counting of individuals from aerial imagery is machine learning, and in particular the development of convolutional neural networks (CNNs), which are highly successful for computer vision tasks. CNNs are a type of deep neural network useful for image classification and recognition, which are composed by two elements: feature extraction and classification. The purpose of feature extraction is to produce feature maps, which is carried out by processes called convolutions^28^. Convolutions consists of applying of a filter that slides over an input image (or other feature map) combining the input value and the filter value to produce another feature map. The process is repeated multiple times with different layers of convolutional filters resulting in multiple layers of feature maps of progressively smaller sizes, where the final layer is a vector of single values, as opposed to tensors of feature maps^29^. Then, the classification part takes this final layer and adds a small number of fully connected layers, similar to a regular feed forward neural network^28,30^. The end of the classification part is a loss function, typically softmax for classification tasks, which provides a predicted probability for each of the target objects. Applications of CNNs to drone imagery have been growing during the past decade^31^. For instance, in koalas (*Phascolarctus cinereus*)^32^, cetaceans^33^, olive ridley sea turtles (*Lepidochelys olivacea*)^34^, kiang (*Equus kiang*)^35^, birds^36–40^ and a set of African terrestrial mammals^41–43^. Depending on the quality of the imagery and the amount of training data, evidence shows that precision and accuracy of detections using CNNs can be high, in some cases better than human counts^25^. As a result, there are opportunities to develop CNNs for a host of different wildlife surveys, including methods to count large mammals in remote locations, such as the challenge caribou pose.

The objectives of this study were to train a CNN to detect and classify caribou from large-scale drone imagery, as most modern CNN architectures are not capable of dealing with huge input images (e.g., mosaics exceeding sizes of 50 k by 50 k pixels). Our aim was to develop an efficient and cost-effective approach to provide accurate and timely information for researchers and wildlife managers. Additionally, in studies where automatic detection and classification algorithms are developed, manual classification is employed for two reasons: first, to train and develop algorithms and secondly for validation^44^. Both processes could be carried out by expert and/or naïve observers (besides citizen science ventures). In this study, we use an expert observer (who was involved in the field data collection) and a team of qualified naïve observers (some of which are experienced image analysts in other contexts) to manually classify detections of different types of caribou (see “Manual counts” section for caribou type details). The experienced observer classifications are used to train and test the CNN model. In addition, annotations of naïve observers are used to mimic a lifelike scenario, where a qualified team of volunteers is employed to generate training data for algorithms in large-scale contexts, from detections and classifications of terrestrial mammals in drone imagery. Thus, our second objective was to compare the CNN model's detections and classifications to the detections and classifications provided by our team of naïve observers. Finally, we discuss the limitations and what is needed to scale up our approach for large-scale studies required to address populations of large terrestrial mammals.

## Methods

### Study area

We conducted drone surveys on 18 July 2016 within the braided delta of the Mast River in Wapusk National Park, Manitoba, Canada (Supplementary Fig. S1.1 online). The study area where imagery was collected is 1 km^2^ (Supplementary Information). It consists primarily of small dwarf willow-dominated (*Salix* sp.) islands (approximately 1–300 m^2^), open graminoid meadows, and river habitat. For an in-depth geophysical and biological description of the study area see^45–48^. The Cape Churchill sub-population present in the study area was estimated in 2937 individuals in 2007 and is part of the Eastern Migratory caribou population, recently designated as Endangered^27^.

### Drone surveys

During drone surveys of nesting common eiders (*Somateria mollissima*)^49^, a herd of caribou moved into the study area and remained bedded down or mostly sedentary for several hours. We opportunistically collected imagery of the entire herd during our eider survey. Flights were performed with small fixed-wing drone (Trimble UX5), which contained a 16.1 MP optical camera in the nadir position. Images of caribou were collected during four flights between 09:08 and 12:41, at altitudes of 120 m (2 flights) and 75 m (2 flights) above ground level (AGL). Following surveys, individual images from each flight were stitched together using Pix4D v. 3.1.22 to create four georeferenced orthomosaic TIFF images (ground sampling distance: 3.7 at 120 m and 2.4 cm at 75 m), which were subsequently used to perform manual and automated counts. For further details such as payload, sensor, data collection, data post-processing, permits, regulations, training, and quality reports of this study, see the Drone Reporting Protocol in the Supplementary Information^50^.

Methods were planned in accordance with the relevant guidelines and regulations. The study was designed considering the potential impacts on target and non-target species. Thus, we flew no less than 75 m above ground level to reduce disturbance on caribou and other biodiversity, as well as were the lowest altitude threshold the Trimble fixed-wing drone could fly. Also, according to national regulations for drone operations, 122 m is the maximum height that we were authorized to fly, so to stay below this threshold we restricted our maximum altitude to 120 m. Data collection and field procedures were authorized by Canadian Wildlife Service Research and Collection Permit 16-MB-SC001, Wapusk National Park WAP-2005-18760 and WAP-2015-18846, UND’s Institutional Animal Care and Use Committee #A3917-01 (protocol 1505-2), UND’s Unmanned Aircraft System Research Compliance Committee reviewed human privacy and data management projects (approved April 10, 2015), and a Special Flight Operations Certificate (File: 5812-11-302, ATS: 17-18-00008,40, RDIMS: 13138829).

### Manual counts

Manual counts of caribou on each of the four mosaics, were performed by six observers. One (AFB) is an experienced observer who participated in the field work activities and is acquainted with the behavior and spatial distribution of this caribou herd. The rest of the five observers (naïve observers) lacked experience with the species, although some are experienced image analysts in other settings. All naïve observers were specifically trained in the counting procedure. To perform the identification and classification, all observers used the platform Open UAS Repository—OUR (https://digitalag.org/our/). OUR is a web-based infrastructure that allows users to upload, share, and collaborate in the analysis of large-scale UAS imagery. Mosaics were uploaded to this repository for the observers to search and count caribou individuals with the aid of a 50 × 50 m grid overlay across the image.

**Figure 1:**
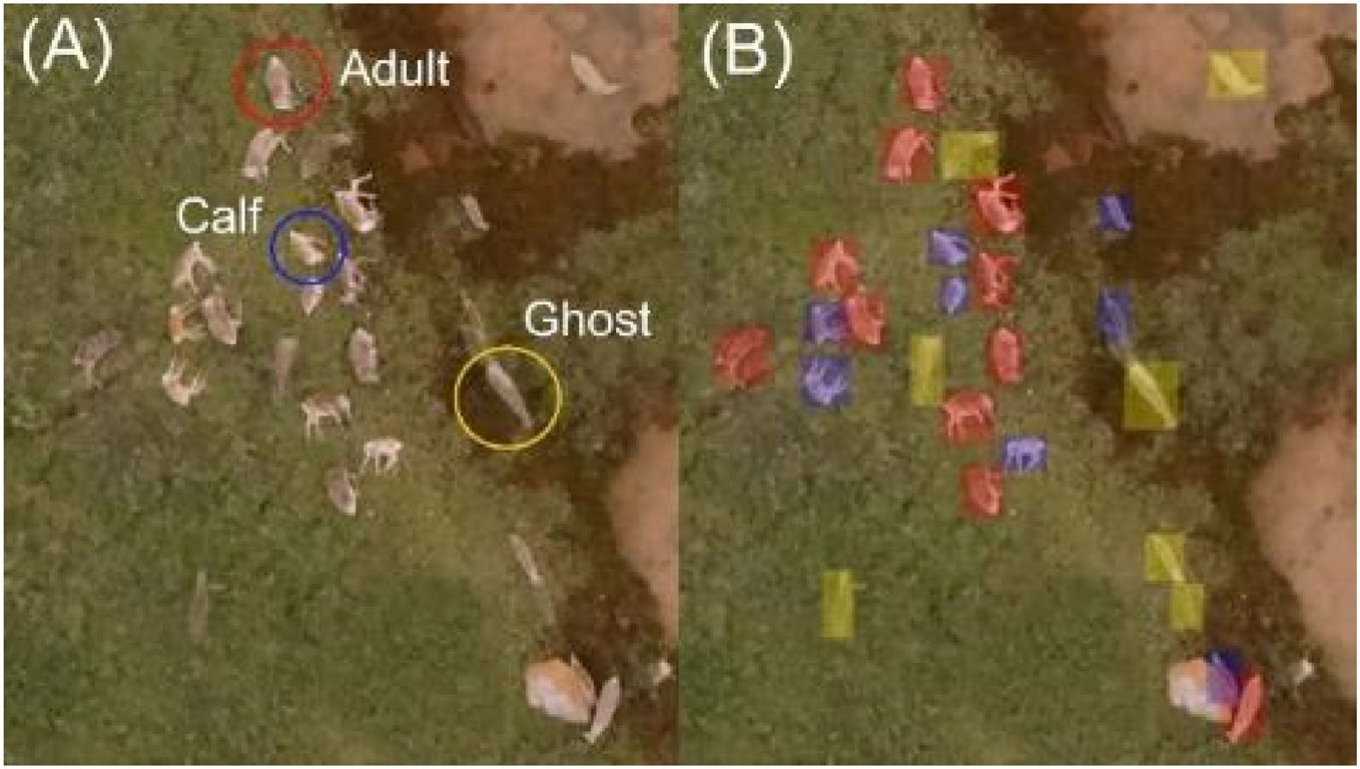
(**A**) Examples of adult (red circle), calf (blue circle), and ghost (yellow circle) caribou that observers classified at the Open UAS Repository—OUR (https://digitalag.org/our/). (**B**) Adults (red rectangles), calves (blue rectangles), and ghosts (yellow rectangles) after the classification in the repository. Figure was assembled using Affinity Designer v 1.9.1.979 (https://affinity.serif.com/).

The counting procedure involved the identification of three types of targets: “adult caribou”, “calf caribou”, and “ghost caribou” that are the product of the image mosaic processing. Although adult caribou were dominant in the images and their body size was variable, calves (smaller individuals) could be distinguished based on their size (Fig. 1a). “Ghosts”, however, could be of either size and appeared as blurred or even transparent in the images (Fig. 1a). Because individuals move during image collection, they become visible in multiple images as “ghosts” that appear in one image but from an overlapping image they are not present, which causes a challenge for the mosaicking process and ultimately the automated recognition algorithms. Thus, we decided to include this category in the classification in order to account for this potential source of error.

During the classification process, the observer used the labeling tool of the Open UAS Repository to draw a rectangle or bounding box surrounding each individual identified (Fig. 1b). Each rectangle contains the actual caribou or ghost including all the pixels and the least possible amount of background (Fig. 1b). After the process of labeling, each classified image box was logged into a text file containing information of the type of label and a list of vertex coordinates (pixel coordinates) of the rectangles for all classified caribou. In addition to labeling, observers were asked to record the time they spent in processing each image, for further comparisons with the automatic detection algorithm.

### Automatic detections: faster-RCNN

We trained a Faster-RCNN (faster region-based convolutional neural network) model^51^ with the goal to be independent of the image resolutions of the sensors or flight characteristics, such as altitude, that impact pixel sizes and ultimately ground sampling distance. To utilize the captured large-scale mosaics as training data, these mosaic images were cut into smaller pieces or “tiles” to train and test the model^52,53^. Tiling is a useful method when computer memory limits the training of large data sets. Thus, the four orthomosaic files (120 m AGL: 845 MB and 980 MB, 75 m AGL: 1.77 GB and 1.88 GB), were tiled to 1000 pixels × 1000 pixels images. Occasionally, as a product of the tiling process, individual caribou (adults, calves, or ghosts) could be cut off and split into two consecutive tiles. To avoid losing or double counting split animals, tiles were overlapped by 100 pixels on the right and lower borders, so that if an animal is located on an edge, it was counted in the following tile (see Supplementary Fig. S2.1 online). Finally, to evaluate the performance of the Faster-RCNN independent of the ground sampling distance, the tiles of the four orthomosaics were shuffled for training.

**Figure 2:**
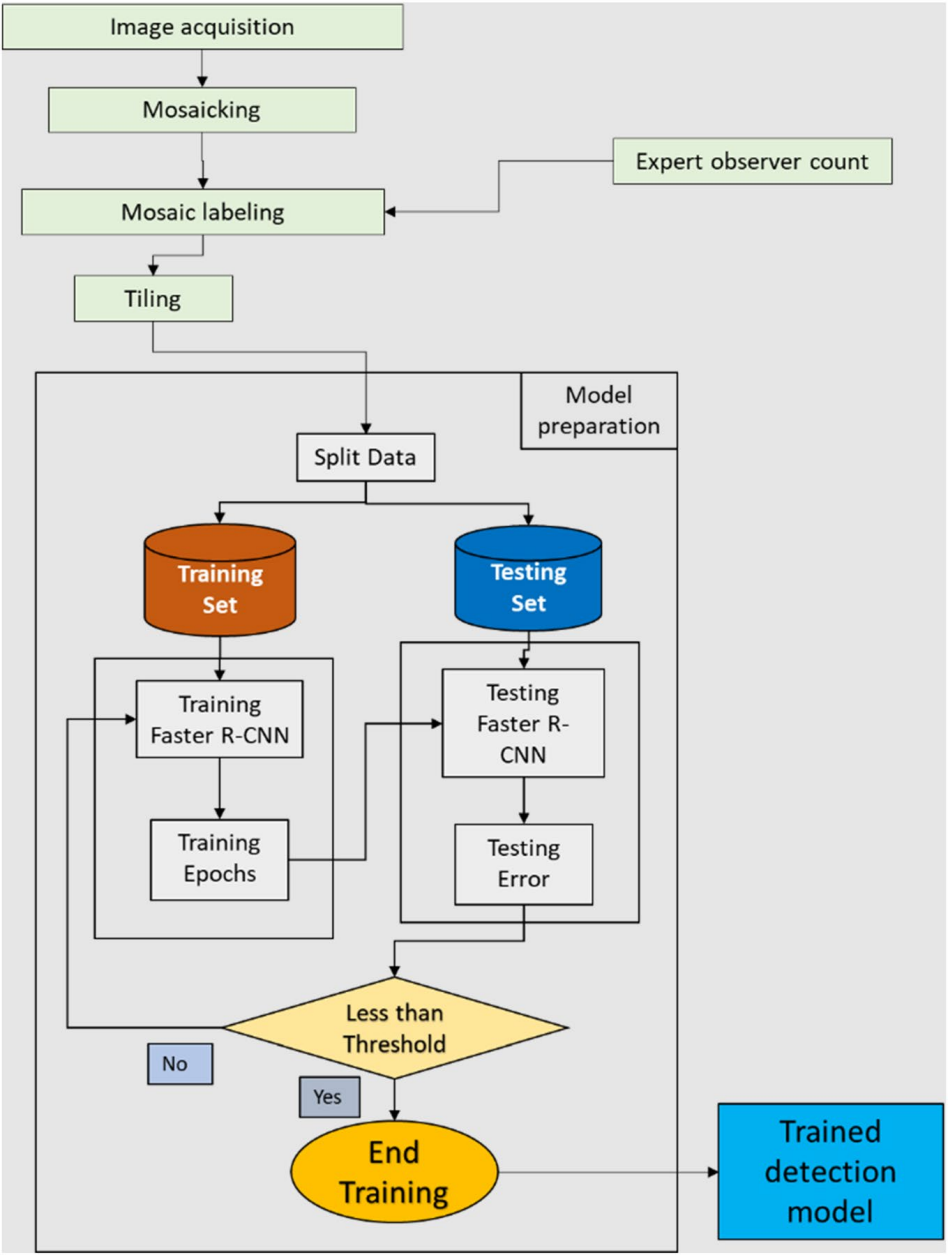
Flowchart of the steps followed to train and test the Faster R-CNN model from the expert observer counts. Results of the trained detection model were then used for comparisons with the experienced observer counts.

A total of 2,148 tiles were produced and split into a training data set of 1607 tiles (75%; Mosaic 1 at 120 m AGL: 271 tiles, Mosaic 2 at 120 m AGL: 288 tiles, Mosaic 3 at 75 m AGL: 548 tiles, and Mosaic 4 at 75 m AGL: 500 tiles) and a testing data set of 541 tiles (25%, including tiles with no caribou on them as negative examples; Mosaic 1 at 120 m AGL: 80 tiles, Mosaic 2 at 120 m AGL: 105 tiles, Mosaic 3 at 75 m AGL: 173 tiles, Mosaic 4 at 75 m AGL: 183 tiles; Fig. 2). The training data set was employed to train the Faster R-CNN model (Fig. 2) using TensorFlow^54^. One NVIDIA GPU and one Intel(R) Xeon(R) CPU at 2.2 GHz were used in the training/testing of the model. The training took a week and performed 20,000 epochs of backpropagation using stochastic gradient decent. During the model training, and at fixed intervals (30 training epochs), the model was assessed using the testing data set. When the training learning curve of the model^55,56^ was flat, the threshold was reached and the training was concluded (see Fig. 2). The model was compared against our experienced observer to determine its performance. Using this approach, we assumed that the experienced observer did not miss any individual and correctly classified all the adults, calves, and ghosts.

The performance of the Faster-RCNN model was evaluated estimating accuracy, precision, and recall. Accuracy was defined as the proportion of true positives in relation to the experienced observer. Precision was defined as the proportion of true positives predicted by the model that were actually correct. Recall was defined as the proportion of true positives in relation to all the relevant elements. Accuracy, precision, and recall were estimated as follows:

1 $$ Accuracy = \frac{true\; positives}{{true\; positives + false\; positives + false \;negatives}} $$

2 $$ Precision = \frac{true \;positives}{{true \;positives + false \;positives}} $$

3 $$ Recall = \frac{true\; positives}{{true \;positives + false \;negatives}} $$

**Figure 3:**
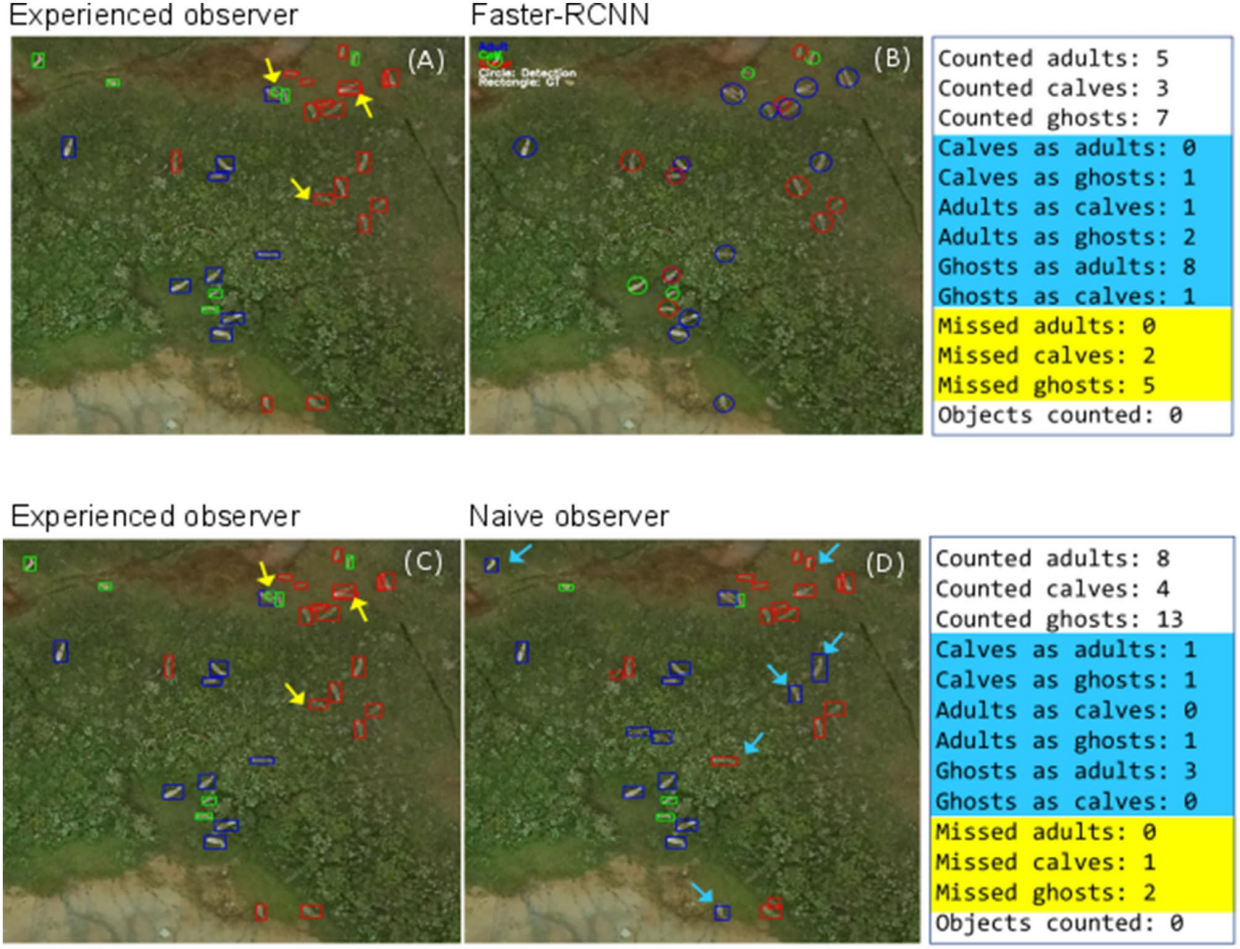
Example of comparison of experienced observer counts (**A**, **C**) with the Faster-RCNN (**B**) and naïve observers (**D**). Blue arrows indicate misclassifications and yellow arrows missed individuals. Note that at the Faster-RCNN image, detection of an adult and a ghost are overlapped. Counted adults are the true positives, misclassifications are false positives, and missed individuals are false negatives. Figure was assembled using Affinity Designer v 1.9.1.979 (https://affinity.serif.com/).

Accuracy, precision and recall were estimated for caribou as a species, and for the caribou categories: adults, calves, and ghosts, and for each of the four mosaic at 120 m and 75 m AGL. Using the testing data set and comparing the tiles classified by the Faster-RCNN model with those classified by our experienced observer, we proceeded to estimate true positives, false positives, and false negatives as follows. First, we counted all the individual caribou identified by the Faster-RCNN model that matched our experienced observer and classified them as true positives. Then, in relation to our experienced observer, we counted the caribou that were missed as false negatives, and those that were misclassified (birds, rocks, trees, and trunks classified as caribou) as false positives (Fig. 3). Second, we counted all the caribou categories separately: adults, calves, and ghosts identified by the Faster-RCNN model that matched our experienced observer and classified them as true positives. Then, we estimated the adults, calves, and ghosts missed by our model as false negatives, and those that were misclassified (calves classified as adults or ghosts, adults as calves or ghosts, ghosts as adults or calves; birds, rocks, trees, and trunks classified as any of these caribou categories) as false positives (Fig. 3). To estimate accuracy, we did not use true negatives because our approach did not consider classifying the absence of caribou in the images, and Faster-RCNN is an object detection model which only draws bounding boxes (detections) on classes of interest. For such models, true negatives are typically not calculated.

### Comparison between the Faster-RCNN model and naïve observers’ classifications

Comparisons between the Faster-RCNN model counts and naïve observers counts were established in relation to our experienced observer. First, from each tile used for testing, we counted all the individual caribou predicted by the Faster-RCNN model output that matched the experienced observer (true positives). Then, we counted the caribou that were missed (false negatives) and those that were misclassified (rocks, birds, trees, and tree trunks; Fig. 3). Secondly, we repeated this procedure incorporating the categories adults, calves, and ghosts. To estimate accuracy, precision, and recall we employed Eqs. 1–3, respectively. In addition, we estimated the percentage difference (i.e., the proportion of detections and classifications in relation to the true positives) to evaluate how the Faster-RCNN model and naïve observers counts compared with the experienced observer. A graphical comparison of the counts in each tile of the experienced observer with the Faster-RCNN model was implemented, and Pearson correlation coefficients estimated. Also, we assessed the amount of time allocated to image classification by all the observers and the time the Faster-RCNN took to produce the outputs, to compare how time-consuming both approaches are. Finally, we evaluated the proportion of misclassifications and missed individuals of the Faster-RCNN model and naïve observers in relation to the true positives for each of the mosaics and caribou class (adults, calves, and ghosts).

## Results

### Faster-RCNN performance

Overall accuracy of the Faster-RCNN model was 80%, precision 90% and recall 88%. The model performed better at higher altitudes, with accuracies between 80 and 88% at 120 m AGL, and between 75 and 76% at 75 m AGL (Table 1). When the performance of the Faster-RCNN model was analyzed by caribou classes and altitudes, the model was more efficient in detecting adults and calves than ghosts at both 120 m AGL mosaics (Table 2). At 75 m AGL, accuracy and precision of adults were higher than calves and ghosts in the first mosaic, although recall of adults was lower than calves and ghosts. In the second 75 m mosaic, the highest accuracy was detected in calves, followed by adults and decreasing markedly in ghosts (Table 2). However, precision for ghosts was higher than adults, although lower than calves in this mosaic, and recall was higher in adults, followed by calves and ghosts (Table 2).

**Table 1:** Comparison between the Faster-RCNN model, expert observer (AFB), and naïve observers counts (raw counts, and true positives: TP, false positives: FP, false negatives: FN, accuracy, precision, recall, and percentage difference: % diff.) of caribou for each of the mosaics at 120 m and 75 m AGL in Wapusk National Park, Manitoba, Canada.

Mosaic	Raw counts	TP	FP	FN	Accuracy	Precision	Recall	% diff
Experienced observer								
Mosaic 1 (120 m)	95							
Mosaic 2 (120 m)	109							
Mosaic 3 (75 m)	63							
Mosaic 4 (75 m)	136							
Model								
Mosaic 1 (120 m)	114	91	16	3	0.88	0.91	0.97	+ 20.0
Mosaic 2 (120 m)	118	101	24	20	0.80	0.95	0.83	+ 8.2
Mosaic 3 (75 m)	103	56	22	1	0.75	0.76	0.98	+ 63.5
Mosaic 4 (75 m)	168	129	24	28	0.76	0.91	0.82	+ 23.5
All observers Avg ± S.D								
Mosaic 1 (120 m)	99.2 ± 9.7	28.0 ± 20.9	3.6 ± 4.1	2.5 ± 2.6	0.81	0.88	0.92	+ 4.0
Mosaic 2 (120 m)	114.4 ± 8.2	33.9 ± 16.6	4.3 ± 3.4	2.1 ± 1.5	0.84	0.89	0.94	+ 4.3
Mosaic 3 (75 m)	67.7 ± 15.9	18.3 ± 16.0	1.9 ± 1.9	1.4 ± 1.7	0.83	0.89	0.93	+ 5.9
Mosaic 4 (75 m)	150.1 ± 14.8	54.1 ± 18.6	4.4 ± 3.7	3.6 ± 5.0	0.84	0.90	0.92	+ 9.3

**Table 2:** Comparison between the Faster-RCNN model, expert observer (AFB), and naïve observers counts (raw counts, true positives: TP, false positives: FP, false negatives: FN, accuracy, precision, recall, and percentage difference: % diff.) of adults, calves, and ghosts caribou for each of the mosaics at 120 m and 75 m AGL in Wapusk National Park, Manitoba, Canada.

Mosaic	Class	Raw counts	TP	FP	FN	Accuracy	Precision	Recall	% diff
Experienced observer									
Mosaic 1 (120 m)	Adults	62							
	Calves	29							
	Ghosts	4							
Mosaic 2 (120 m)	Adults	59							
	Calves	25							
	Ghosts	25							
Mosaic 3 (75 m)	Adults	44							
	Calves	9							
	Ghosts	10							
Mosaic 4 (75 m)	Adults	77							
	Calves	25							
	Ghosts	34							
Faster RCNN model									
Mosaic 1 (120 m)	Adults	77	65	10	0	0.87	0.87	1.00	+ 19.5
	Calves	27	24	1	2	0.89	0.96	0.92	− 7.4
	Ghosts	10	6	5	1	0.50	0.55	0.86	+ 140.0
Mosaic 2 (120 m)	Adults	70	51	13	5	0.74	0.80	0.91	+ 15.7
	Calves	26	19	5	3	0.70	0.79	0.86	+ 3.8
	Ghosts	22	16	6	12	0.47	0.73	0.57	− 12.0
Mosaic 3 (75 m)	Adults	69	48	12	1	0.79	0.80	0.98	+ 36.2
	Calves	19	11	6	0	0.65	0.65	1.00	+ 52.6
	Ghosts	15	12	4	0	0.75	0.75	1.00	+ 33.3
Mosaic 4 (75 m)	Adults	102	78	17	0	0.82	0.82	1.00	+ 24.5
	Calves	29	22	2	2	0.85	0.92	0.92	+ 13.8
	Ghosts	37	31	5	26	0.50	0.86	0.54	+ 8.1
All observers (Avg ± S.D.)									
Mosaic 1 (120 m)	Adults	56.6 ± 8.6	56.4 ± 8.7	3.2 ± 1.8	3.8 ± 3.8	0.89	0.94	0.93	− 9.7
	Calves	27.6 ± 3.0	24.8 ± 2.9	2.8 ± 2.4	2.6 ± 2.3	0.83	0.89	0.91	− 6.9
	Ghosts	11.2 ± 7.9	5.8 ± 3.1	5.4 ± 6.5	1.2 ± 1.1	0.50	0.59	0.83	+ 63.6
Mosaic 2 (120 m)	Adults	60.6 ± 5.9	55.0 ± 6.5	6.0 ± 1.9	2.4 ± 2.1	0.87	0.90	0.96	+ 1.6
	Calves	25.0 ± 3.9	21.2 ± 2.7	3.8 ± 2.8	1.6 ± 1.3	0.81	0.85	0.93	0.0
	Ghosts	28.8 ± 10.1	25.6 ± 8.2	3.2 ± 4.5	2.4 ± 1.1	0.83	0.91	0.91	+ 13.7
Mosaic 3 (75 m)	Adults	41.5 ± 4.4	39.8 ± 4.5	1.2 ± 0.8	1.4 ± 1.3	0.94	0.97	0.97	− 6.8
	Calves	11.4 ± 3.0	7.4 ± 1.5	4.0 ± 2.6	0.4 ± 0.5	0.64	0.67	0.95	+ 18.2
	Ghosts	9.2 ± 3.5	7.6 ± 2.5	1.6 ± 2.1	2.4 ± 2.3	0.67	0.86	0.76	− 10.0
Mosaic 4 (75 m)	Adults	66.8 ± 8.3	63.0 ± 7.6	3.8 ± 2.5	6.2 ± 6.5	0.86	0.94	0.91	− 12.9
	Calves	27.8 ± 3.9	24.6 ± 2.6	3.2 ± 2.9	1.0 ± 1.0	0.86	0.89	0.96	+ 10.7
	Ghosts	54.8 ± 16.6	47.8 ± 14.7	7.0 ± 4.8	3.8 ± 5.2	0.80	0.87	0.93	+ 38.2

### Faster-RCNN versus manual counts

**Figure 4:**
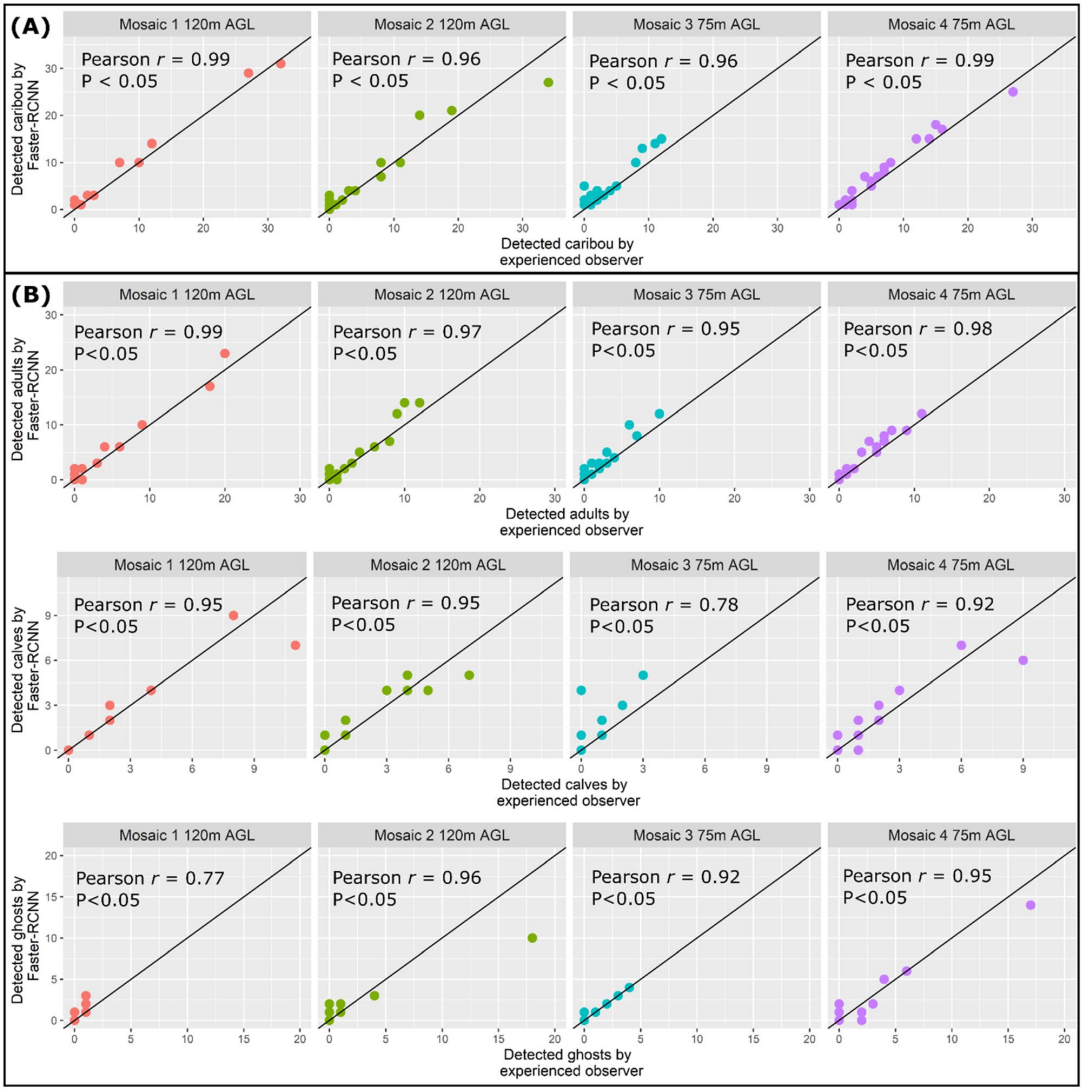
Comparison between (**A**) the individual caribou counts and (**B**) caribou adults, calves, and ghosts of the experienced observer and the Faster-RCNN per tile for each mosaic. The diagonal 1:1 line indicates a perfect fit of the experienced observer and Faster-RCNN counts. Plots were created using R v 4.1.3 (R Core Team, 2022) and figure assembled using Affinity Designer v 1.9.1.979 (https://affinity.serif.com/).

The counts of the Faster-RCNN and experienced observer per tile for each of the mosaics showed high correlation at 120 m and 75 m (Fig. 4). Overall, most of the detections of the Faster-RCNN model seem overestimated in relation to the experienced observer (Fig. 4), and the percentage difference between both confirms an overestimation between a minimum of + 8.2% (at 120 m) and maximum of + 63.5% (at 75 m, Table 1).

Faster-RCNN and the experienced observer counts per tile and per caribou class for each of the mosaics, showed in general high correlations for adults, calves, and ghosts (Fig. 4). In most cases, the Faster-RCNN overestimated the count of adults, calves, and ghosts (Fig. 4, Table 2). The percentage difference for adults ranged between + 19.5% (at 75 m) and + 36.2% (at 75 m). For calves, this percentage oscillated between − 7.4% (at 120 m) and + 52.6% (75 m), and for ghosts, between -12.0% (120 m) and + 140.0% (120 m) (Table 2).

Overall, naïve observers showed relatively high accuracy and precision in each of the mosaics (Table 1); when adults, calves, and ghosts were analyzed independently, accuracy and precision ranges were more variable, especially because ghosts were detected and classified with higher error (Table 2). Percentage difference at 120 m ranged between − 9.7% and + 63.6%, and at 75 m varied between − 12.9% and + 38.2% (Table 1). Adults percentage difference varied between − 12.9% at 75 m and + 1.6% at 120 m, while calves varied between − 26.9% at 120 m and + 18.2% at 75 m. Ghost was the most variable category with percentage differences between − 10.0% at 75 m and − 63.6% at 120 m.

In relation to time spent by the human observers for the classification of the images, it took on average 121.6 ± 45.9 min for the first 75 m mosaic, 103.8 ± 66.1 min for the second 75 m mosaic, 59.2 ± 20.3 min for the third mosaic at 120 m, and 51.9 ± 42.5 min for the last 120 m mosaic. Not considering the training phase of the Faster-RCNN model that took 1 week, the time that it took the model to process the testing set was 19.8 min.

### Misclassifications and missing individuals

In the first 120 m mosaic, the Faster-RCNN missed less individuals and proportions of adults, calves, and ghosts than the naïve observers (Table 3, Supplementary Fig. S2.2 online). In the second 120 m mosaic, however, naïve observers detected more ghosts than the Faster-RCNN model, although calves and adults were better detected by the model (Table 3, Supplementary Fig. S2.2 online). In the first mosaic at 75 m, the Faster-RCNN model did not miss calves and ghosts and missed only one adult (Table 3, Supplementary Fig. S2.2 online). In the second mosaic at 75 m, the Faster-RCNN model did not miss adults, detected more calves and a much lower proportion of ghosts than observers (Table 3, Supplementary Fig. S2.2 online).

**Table 3:** Number and proportion of caribou adults, calves, and ghosts that were missed by the Faster-RCNN and the pool of naïve observers (mean ± SD) per mosaic.

Mosaic	Class	Missing	Proportion missing
Faster-RCNN model			
Mosaic 1 (120 m)	Adults	0	0.00
	Calves	2	0.08
	Ghosts	1	0.14
Mosaic 2 (120 m)	Adults	5	0.09
	Calves	3	0.14
	Ghosts	12	0.43
Mosaic 3 (75 m)	Adults	1	0.02
	Calves	0	0.00
	Ghosts	0	0.00
Mosaic 4 (75 m)	Adults	0	0.00
	Calves	2	0.08
	Ghosts	26	0.46
Naïve observers			
Mosaic 1 (120 m)	Adults	3.8 ± 3.7	0.07
	Calves	2.6 ± 2.3	0.09
	Ghosts	1.2 ± 1.1	0.20
Mosaic 2 (120 m)	Adults	2.4 ± 2.1	0.04
	Calves	1.6 ± 1.3	0.07
	Ghosts	2.4 ± 1.1	0.09
Mosaic 3 (75 m)	Adults	1.4 ± 1.3	0.03
	Calves	0.4 ± 0.5	0.05
	Ghosts	2.4 ± 2.3	0.24
Mosaic 4 (75 m)	Adults	6.2 ± 6.5	0.09
	Calves	1.0 ± 1.0	0.04
	Ghosts	3.8 ± 5.3	0.09

Faster-RCNN classified caribou better than the pool of observers in the first 120 m mosaic. Adults classified as ghosts, was the misclassification with the highest proportion for both, the Faster-RCNN model and observers (Table 4, Supplementary Fig. S2.3 online). In the second 120 m mosaic, Faster-RCNN classified equally or better than the pool of naïve observers, except for ghosts classified as adults that was higher for the model (Table 4, Supplementary Fig. S2.3 online). At 75 m, misclassifications of the Faster-RCNN model were overall lower than the observer’s misclassifications (Table 4, Supplementary Fig. S2.3 online). However, in the first mosaic the model misclassified more calves as adults and ghosts as calves. In the second 75 m mosaic, the model misclassified more calves as ghosts, and ghosts as adults (Table 4, Supplementary Fig. S2.3 online).

**Table 4:** Counts and proportion of misclassifications of adults, calves, and ghosts of the Faster-RCNN and naïve observers (mean ± SD) per mosaic.

Class	Mosaic	Faster-RCNN counts	Faster-RCNN proportions	Naïve observers counts	Naïve observers proportions
Adult as calf	Mosaic 1 120 m AGL	1	0.04	2.6 ± 1.9	0.09
Adult as ghost	Mosaic 1 120 m AGL	3	0.33	4.2 ± 5.5	0.34
Calf as adult	Mosaic 1 120 m AGL	2	0.03	2.4 ± 1.3	0.04
Calf as ghost	Mosaic 1 120 m AGL	1	0.14	1.2 ± 1.3	0.15
Ghost as adult	Mosaic 1 120 m AGL	0	0.00	0.8 ± 8.3	0.02
Ghost as calf	Mosaic 1 120 m AGL	0	0.00	0.2 ± 0.4	0.01
Adult as calf	Mosaic 2 120 m AGL	3	0.14	3.2 ± 2.4	0.13
Adult as ghost	Mosaic 2 120 m AGL	2	0.11	2.2 ± 2.8	0.06
Calf as adult	Mosaic 2 120 m AGL	3	0.06	2.8 ± 0.8	0.05
Calf as ghost	Mosaic 2 120 m AGL	1	0.06	1.0 ± 1.7	0.03
Ghost as adult	Mosaic 2 120 m AGL	9	0.15	3.2 ± 1.9	0.06
Ghost as calf	Mosaic 2 120 m AGL	1	0.05	0.6 ± 0.5	0.03
Adult as calf	Mosaic 3 75 m AGL	0	0.00	3.4 ± 2.4	0.29
Adult as ghost	Mosaic 3 75 m AGL	1	0.08	1.2 ± 1.8	0.10
Calf as ghost	Mosaic 3 75 m AGL	0	0.00	0.4 ± 0.5	0.05
Calf as adult	Mosaic 3 75 m AGL	2	0.04	0.2 ± 0.4	0.0
Ghost as calf	Mosaic 3 75 m AGL	1	0.08	0.6 ± 0.9	0.08
Ghost as adult	Mosaic 3 75 m AGL	0	0.00	1.0 ± 1.0	0.03
Adult as calf	Mosaic 4 75 m AGL	1	0.04	2.0 ± 1.2	0.07
Adult as ghost	Mosaic 4 75 m AGL	3	0.09	6.2 ± 4.1	0.10
Calf as adult	Mosaic 4 75 m AGL	2	0.03	1.8 ± 1.9	0.03
Calf as ghost	Mosaic 4 75 m AGL	1	0.03	0.8 ± 1.3	0.01
Ghost as adult	Mosaic 4 75 m AGL	4	0.05	2.0 ± 0.7	0.03
Ghost as calf	Mosaic 4 75 m AGL	1	0.04	1.2 ± 1.8	0.04

Naïve observers did not misclassify caribou as objects i.e., trees, trunks, rocks, or birds. However, the Faster-RCNN model did misclassify caribou as objects in all the mosaics. At 120 m, 9 objects were misclassified as adults, 1 as a calf, and 1 as a ghost. At 75 m, 22 objects were misclassified as adults, 5 as calves, and 4 as ghosts.

## Discussion

To the best of our knowledge, we present one of the first attempts to employ automated detection of large mammals from drone-based imagery in North America. We have developed a method for training Faster-RCNN models given large-scale mosaic imagery to classify and count caribou adults, calves, and ghosts independent of the altitude and ground sampling distance of the collected imagery. After having compared the image detections and classifications of the Faster-RCNN model with those of an experienced observer on the same images, we noticed that the automatic detection and classification performance could be promising for future implementations. When the analysis was performed by mosaic and caribou class, the Faster-RCNN model performance was also promising, in some cases it accomplished better outcomes than the naïve observers. However, ghost was the category which detection and classification were both challenging by the Faster-RCNN and naïve observers. Adults and calves in some cases were better detected and classified by the Faster-RCNN model than the naïve observers. However, a high proportion of adults were misclassified as calves in all the mosaics, mostly by some naïve observers rather than the Faster-RCNN model. This study suggests that there is a need to improve consistency among observers to better classify groups, required to train models accurately in large-scale studies. These types of studies are also challenged by double counting of individuals, a problem that needs to be overcome. Our study found that having trained the model from images with different ground sampling distances, detection and classification of caribou is satisfactory, opening new promising avenues for the implementation of large-scale studies and monitoring.

In applied contexts, the benefits of drone technology could be challenged by the high amount of information collected by the sensors, which artificial intelligence is attempting to unravel. Given these extensive data sets, especially from highly mobile species as the one analyzed in this study, practitioners could be benefitted by using teams of human observers as ground truth for labeling and model training. In this scenario, a minimum level of consistency is desirable for successfully training algorithms from multiple operators^57^, because accuracy of a model could be undermined by the high uncertainty of the observer annotations^58^. Our study found that detection and classification varied among observers, which opens questions on how to minimize this variability for further implementation of artificial intelligence in large-scale applied settings. Further, we propose the following actions when designing surveys to improve the quality of training data: (a) the expansion of the training phase and regular assessments of observers’ asymptotic learning curves; (b) working as a group to allow for collective and common learning conducive to a more standardized experience; (c) to provide observers with the same set of tools, such as screens sizes and resolutions, similar timelines with appropriate resting times, and other standard working conditions like light, temperature, and ventilation; and finally (d) aid observers with alternative tools such as GPS or radio tracked caribou to verify true positive detections. This way we might be able to account for inter-observer variation and train models from multiple observers in large-scale situations.

One benefit of mosaicking is the potential reduction of double counting when animals move across the landscape, since overlapped areas in the imagery are excluded^8,59^; but the process is not perfect, and ghosts emerge are one drawback that we aimed to account for with our classification system. It is noteworthy that our Faster-RCNN model had issues detecting ghosts in two mosaics, one at 120 m and one at 75 m AGL, which similarly happened with the naïve observers. The model had also difficulties to correctly classify ghosts that were mostly confounded with adults, although did better than some naïve observers. Considering that the movements of the caribou herd analyzed in this study were relatively slow, the question of if mosaicking reduce double counting would work with highly mobile species, arise and might be further assessed. To avoid the presence of ghosts in mosaics, it could be useful to use the original raw images or strip sampling as an alternative^59^, although additional efforts should be allocated to reduce the number of double-counts. For instance, flying more than one drone simultaneously, similar to employ multiple field observers, could reduce double counts, although it could be costly. It may also be helpful to incorporate object tracking components from video footage into the CNN analysis method, to reduce double counts of the same individuals. In any case, it is important that flight plans consider minimizing behavioral responses to reduce the chances that animals do not move in reaction to the aircraft^59,60^. Moreover, if we could design surveys to reduce double counting close to zero, we could be able to explore the use of hierarchical models to detect and classify individuals. It has been proposed that *N*-mixture models is a good method to estimate abundance from aerial imagery^59^, although these models are very sensitive to violations of assumptions, i.e., observers do not double count individuals, counts are independent binomial random variables, detection probabilities are constant, abundance is random and independent, and the population is closed^61,62^. Other approaches like a modification of the Horvitz–Thompson method^63^ that combines generalized linear and additive models for overall probability of detection, false detection, and duplicate detection have been proposed as better alternatives to *N*-mixture models^64^. This could be a promising avenue to couple models that account for imperfect detection to train neural networks, especially in contexts where data sets are becoming large and difficult to process by human observers.

An algorithm able to learn and classify animals from imagery taken at different altitudes or ground sampling distances could be an advantage for generalizable models^65^, especially useful for researchers and practitioners. Our Faster-RCNN model could be able to satisfactorily detect and classify caribou at both altitudes with different ground sampling distances. This might open further avenues to overcome difficulties that prevent combining different sources of data, especially when dealing with broadly distributed species. For example, different types of airspaces that constrain flights at certain altitudes could vary between countries and regions. Additionally, access to standardized platforms and sensors for long term and large-scale studies is a challenge, which could be overcome with algorithms like ours, potentially independent of ground sampling distances. Some successful examples of this approach are present in the literature for the detection of conifers^66^, crops^67^, and large mammals^15^. To achieve that, *inter alia*, we needed to assess what are the limits for algorithms to be trained with a range of ground sampling distances, able to accurately classify targets; in addition to evaluations under different whether conditions, backgrounds, and species. Ultimately, we could be able to generalize and optimize resources and data, to leverage the application of this technology for studying and managing wildlife.

To successfully apply drone technology to large-scale studies of large mammals, we need to scale up flights to larger landscapes rather than smaller areas. However, there are still technical and logistic limitations related to the use of beyond visual line of sight platforms (BVLOS) that facilitate larger areas surveys. A few examples of BVLOS use have been carried out in wildlife ecology, mostly in marine settings. For instance, Hodgson et al.^68^ assessed the detection probability of a BVLOS drone platform to detect humpback whales (*Megaptera novaeangliae*) in Australia. Similarly, Ferguson et al.^69^ evaluated the performance of images taken from a drone platform in relation to direct surveys and imagery from manned aircraft, to detect and count marine mammals in Alaska; BVLOS platforms are promising although still expensive and less efficient than human observers onboard occupied aircrafts, authors concluded. Isolated marine biodiversity such as marine mammals, seabirds, and tundra communities have successfully been surveyed on King George island in Antarctica using BVLOS technology^70^. Nevertheless, a bigger problem BVLOS surveys have, would be the sheer amount of data collected, and concordantly with our findings, manual counts of wildlife are not scalable due to time restrictions^71^.

## Supplementary Information

Supplementary Information.

## Data Availability

The data that support the findings of this study are available from the corresponding author upon request.

## References

[1] Chapman A (2014). It’s okay to call them drones. J. Unmanned Veh. Syst..

[2] Chabot D, Hodgson AJ, Hodgson JC, Anderson K (2022). ‘Drone’: Technically correct, popularly accepted, socially acceptable. Drone Syst. Appl..

[3] Chabot D, Bird DM (2015). Wildlife research and management methods in the 21st century: Where do unmanned aircraft fit in?. J. Unmanned Veh. Syst..

[4] Christie KS, Gilbert SL, Brown CL, Hatfield M, Hanson L (2016). Unmanned aircraft systems in wildlife research: Current and future applications of a transformative technology. Front. Ecol. Environ..

[5] Whitehead K, Hugenholtz CH (2014). Remote sensing of the environment with small unmanned aircraft systems (UASs), part 1: A review of progress and challenges. J. Unmanned Veh. Syst..

[6] Barnas A (2018). Evaluating behavioral responses of nesting lesser snow geese to unmanned aircraft surveys. Ecol. Evol..

[7] Mulero-Pázmány M (2017). Unmanned aircraft systems as a new source of disturbance for wildlife: A systematic review. PLoS ONE.

[8] Linchant J, Lisein J, Semeki J, Lejeune P, Vermeulen C (2015). Are unmanned aircraft systems (UAS s) the future of wildlife monitoring? A review of accomplishments and challenges. Mammal Rev..

[9] Whitehead K (2014). Remote sensing of the environment with small unmanned aircraft systems (UASs), part 2: Scientific and commercial applications. J. Unmanned Veh. Syst..

[10] Barasona JA (2014). Unmanned aircraft systems for studying spatial abundance of ungulates: Relevance to spatial epidemiology. PLoS ONE.

[11] Chrétien LP, Théau J, Ménard P (2015). Wildlife multispecies remote sensing using visible and thermal infrared imagery acquired from an unmanned aerial vehicle (UAV). Int. Arch. Photogramm. Remote Sens. Spat. Inf. Sci..

[12] Guo X (2018). Application of UAV remote sensing for a population census of large wild herbivores—Taking the headwater region of the yellow river as an example. Remote Sens..

[13] Hu J, Wu X, Dai M (2020). Estimating the population size of migrating Tibetan antelopes *Pantholops hodgsonii* with unmanned aerial vehicles. Oryx.

[14] Mulero-Pázmány M, Stolper R, Van Essen LD, Negro JJ, Sassen T (2014). Remotely piloted aircraft systems as a rhinoceros anti-poaching tool in Africa. PLoS ONE.

[15] Rey N, Volpi M, Joost S, Tuia D (2017). Detecting animals in African Savanna with UAVs and the crowds. Remote Sens. Environ..

[16] Schroeder NM, Panebianco A, Gonzalez Musso R, Carmanchahi P (2020). An experimental approach to evaluate the potential of drones in terrestrial mammal research: A gregarious ungulate as a study model. R. Soc. Open Sci..

[17] Su X (2018). Using an unmanned aerial vehicle (UAV) to study wild yak in the highest desert in the world. Int. J. Remote Sens..

[18] Vermeulen C, Lejeune P, Lisein J, Sawadogo P, Bouché P (2013). Unmanned aerial survey of elephants. PLoS ONE.

[19] Mallory ML (2018). Financial costs of conducting science in the Arctic: Examples from seabird research. Arct. Sci..

[20] Sasse DB (2003). Job-related mortality of wildlife workers in the United States, 1937–2000. Wildl. Soc. Bull..

[21] Loarie SR, Joppa LN, Pimm SL (2007). Satellites miss environmental priorities. Trends Ecol. Evol..

[22] IUCN. The IUCN Red List of Threatened Species. *IUCN Red List of Threatened Species* https://www.iucnredlist.org/en (2021).

[23] Mech, L. D. & Barber, S. M. *A critique of wildlife radio-tracking and its use in National Parks: a report to the National Park Service*. (2002).

[24] Patterson C, Koski W, Pace P, McLuckie B, Bird DM (2015). Evaluation of an unmanned aircraft system for detecting surrogate caribou targets in Labrador. J. Unmanned Veh. Syst..

[25] Hodgson JC (2018). Drones count wildlife more accurately and precisely than humans. Methods Ecol. Evol..

[26] Seymour AC, Dale J, Hammill M, Halpin PN, Johnston DW (2017). Automated detection and enumeration of marine wildlife using unmanned aircraft systems (UAS) and thermal imagery. Sci. Rep..

[27] COSEWIC. *COSEWIC assessment and status report on the caribou (Rangifer tarandus) eastern migratory population, Torngat mountain population in Canada*. (COSEWIC, Committee on the Status of Endangered Wildlife in Canada, 2017).

[28] Albawi, S., Mohammed, T. A. & Al-Zawi, S. Understanding of a convolutional neural network. in *2017 international conference on engineering and technology (ICET)* 1–6 (IEEE, 2017).

[29] Gu J (2018). Recent advances in convolutional neural networks. Pattern Recognit..

[30] Teuwen, J. & Moriakov, N. Convolutional neural networks. in *Handbook of medical image computing and computer assisted intervention* 481–501 (Elsevier, 2020).

[31] Corcoran E, Winsen M, Sudholz A, Hamilton G (2021). Automated detection of wildlife using drones: Synthesis, opportunities and constraints. Methods Ecol. Evol..

[32] Corcoran E, Denman S, Hanger J, Wilson B, Hamilton G (2019). Automated detection of koalas using low-level aerial surveillance and machine learning. Sci. Rep..

[33] Gray PC (2019). Drones and convolutional neural networks facilitate automated and accurate cetacean species identification and photogrammetry. Methods Ecol. Evol..

[34] Gray PC (2019). A convolutional neural network for detecting sea turtles in drone imagery. Methods Ecol. Evol..

[35] Peng J (2020). Wild animal survey using UAS imagery and deep learning: modified Faster R-CNN for kiang detection in Tibetan Plateau. ISPRS J. Photogramm. Remote Sens..

[36] Borowicz A (2018). Multi-modal survey of Adélie penguin mega-colonies reveals the Danger Islands as a seabird hotspot. Sci. Rep..

[37] Francis RJ, Lyons MB, Kingsford RT, Brandis KJ (2020). Counting mixed breeding aggregations of animal species using drones: Lessons from waterbirds on semi-automation. Remote Sens..

[38] Santangeli A (2020). Integrating drone-borne thermal imaging with artificial intelligence to locate bird nests on agricultural land. Sci. Rep..

[39] Bowley C, Mattingly M, Barnas A, Ellis-Felege S, Desell T (2019). An analysis of altitude, citizen science and a convolutional neural network feedback loop on object detection in unmanned aerial systems. J. Comput. Sci..

[40] Bowley, C., Mattingly, M., Barnas, A., Ellis-Felege, S. & Desell, T. Detecting wildlife in unmanned aerial systems imagery using convolutional neural networks trained with an automated feedback loop. in *International Conference on Computational Science* 69–82 (Springer, 2018).

[41] Delplanque A, Foucher S, Lejeune P, Linchant J, Théau J (2021). Multispecies detection and identification of African mammals in aerial imagery using convolutional neural networks. Remote Sens. Ecol. Conserv..

[42] Eikelboom JAJ (2019). Improving the precision and accuracy of animal population estimates with aerial image object detection. Methods Ecol. Evol..

[43] Kellenberger B, Marcos D, Tuia D (2018). Detecting mammals in UAV images: Best practices to address a substantially imbalanced dataset with deep learning. Remote Sens. Environ..

[44] Hooge ITC, Niehorster DC, Nyström M, Andersson R, Hessels RS (2018). Is human classification by experienced untrained observers a gold standard in fixation detection?. Behav. Res. Methods.

[45] Barnas AF, Darby BJ, Vandeberg GS, Rockwell RF, Ellis-Felege SN (2019). A comparison of drone imagery and ground-based methods for estimating the extent of habitat destruction by lesser snow geese (*Anser caerulescens caerulescens*) in La Pérouse Bay. PLoS ONE.

[46] Brook RK, Kenkel NC (2002). A multivariate approach to vegetation mapping of Manitoba’s Hudson Bay Lowlands. Int. J. Remote Sens..

[47] Shilts, W. W., Aylsworth, J. M., Kaszycki, C. A., Klassen, R. A. & Graf, W. L. Canadian shield. in *Geomorphic Systems of North America* vol. 2 119–161 (Geological Society of America Boulder, Colorado, 1987).

[48] Barnas AF, Felege CJ, Rockwell RF, Ellis-Felege SN (2018). A pilot (less) study on the use of an unmanned aircraft system for studying polar bears (*Ursus maritimus*). Polar Biol..

[49] Ellis-Felege SN (2021). Nesting common eiders (*Somateria mollissima*) show little behavioral response to fixed-wing drone surveys. J. Unmanned Veh. Syst..

[50] Barnas AF (2020). A standardized protocol for reporting methods when using drones for wildlife research. J. Unmanned Veh. Syst..

[51] Ren S, He K, Girshick R, Sun J (2016). Faster R-CNN: Towards real-time object detection with region proposal networks. Adv. Neural Inf. Process. Syst..

[52] Chen, T., Xu, B., Zhang, C. & Guestrin, C. Training Deep Nets with Sublinear Memory Cost. *ArXiv160406174 Cs* (2016).

[53] Pinckaers, H. & Litjens, G. Training convolutional neural networks with megapixel images. *ArXiv180405712 Cs* (2018). 10.1109/TPAMI.2020.3019563 32845835

[54] Abadi, M. *et al.* TensorFlow: Large-scale machine learning on heterogeneous systems. (2015).

[55] Janocha, K. & Czarnecki, W. M. On loss functions for deep neural networks in classification. *ArXiv Prepr. ArXiv170205659*. (2017).

[56] Murata N, Yoshizawa S, Amari S (1992). Learning curves, model selection and complexity of neural networks. Adv. Neural Inf. Process. Syst..

[57] Hänsch, R. & Hellwich, O. The truth about ground truth: Label noise in human-generated reference data. in *IGARSS 2019–2019 IEEE International Geoscience and Remote Sensing Symposium* 5594–5597 (IEEE, 2019).

[58] Bowler E, Fretwell PT, French G, Mackiewicz M (2020). Using deep learning to count albatrosses from space: Assessing results in light of ground truth uncertainty. Remote Sens..

[59] Brack IV, Kindel A, Oliveira LFB (2018). Detection errors in wildlife abundance estimates from Unmanned Aerial Systems (UAS) surveys: Synthesis, solutions, and challenges. Methods Ecol. Evol..

[60] Jagielski PM (2022). The utility of drones for studying polar bear behaviour in the Canadian Arctic: Opportunities and recommendations. Drone Syst. Appl..

[61] Williams PJ, Hooten MB, Womble JN, Bower MR (2017). Estimating occupancy and abundance using aerial images with imperfect detection. Methods Ecol. Evol..

[62] Link WA, Schofield MR, Barker RJ, Sauer JR (2018). On the robustness of N-mixture models. Ecology.

[63] Horvitz DG, Thompson DJ (1952). A generalization of sampling without replacement from a finite universe. J. Am. Stat. Assoc..

[64] Corcoran E, Denman S, Hamilton G (2020). New technologies in the mix: Assessing N-mixture models for abundance estimation using automated detection data from drone surveys. Ecol. Evol..

[65] Lunga D, Arndt J, Gerrand J, Stewart R (2021). ReSFlow: A remote sensing imagery data-flow for improved model generalization. IEEE J. Sel. Top. Appl. Earth Obs. Remote Sens..

[66] Fromm M, Schubert M, Castilla G, Linke J, McDermid G (2019). Automated detection of conifer seedlings in drone imagery using convolutional neural networks. Remote Sens..

[67] Velumani K (2021). Estimates of maize plant density from UAV RGB images using Faster-RCNN detection model: Impact of the spatial resolution. Plant Phenomics.

[68] Hodgson A, Peel D, Kelly N (2017). Unmanned aerial vehicles for surveying marine fauna: Assessing detection probability. Ecol. Appl..

[69] Ferguson MC (2018). Performance of manned and unmanned aerial surveys to collect visual data and imagery for estimating arctic cetacean density and associated uncertainty. J. Unmanned Veh. Syst..

[70] Zmarz A (2018). Application of UAV BVLOS remote sensing data for multi-faceted analysis of Antarctic ecosystem. Remote Sens. Environ..

[71] Lyons MB (2019). Monitoring large and complex wildlife aggregations with drones. Methods Ecol. Evol..

