# Supplementary material for: Artificial intelligence for automated detection of large mammals creates path to upscale drone surveys
Source: Sci Rep. 2023 Jan 18;13:947. doi: 10.1038/s41598-023-28240-9 (PMC9849265; doi:10.1038/s41598-023-28240-9)

1. Drone Reporting Protocol for Artificial intelligence for automated detection of large mammals creates path to upscale drone surveys

Produced by Andrew Barnas[1,2] and Susan Ellis-Felege[1], July 2020

[1]Department of Biology, University of North Dakota, 10 Cornell Street, Stop 9019, Grand Forks, ND 58202, USA
[2]School of Environmental Studies, University of Victoria, Victoria, V8W 2Y2, British Columbia, Canada

1.1 Project Overview

The objective of this research was to investigate automated methods of detecting individual caribou (*Rangifer tarandus*) from drone imagery. To do this, we conducted drone surveys over a small herd of caribou along the northwestern border of Wapusk National Park, Manitoba, Canada (Figure S1) as part of a larger effort focused on surveying a common eider (*Somateria mollissima*) nesting colony. We collected still RGB imagery of caribou on 18 July 2016 with a fixed wing aircraft and created georeferenced orthomosaics for use in manual and automated detection methods. In the following sections we describe the technical specifications of the drone platform and sensors used, as well as additional details on image collection and processing prior to manual image review and automated methods.

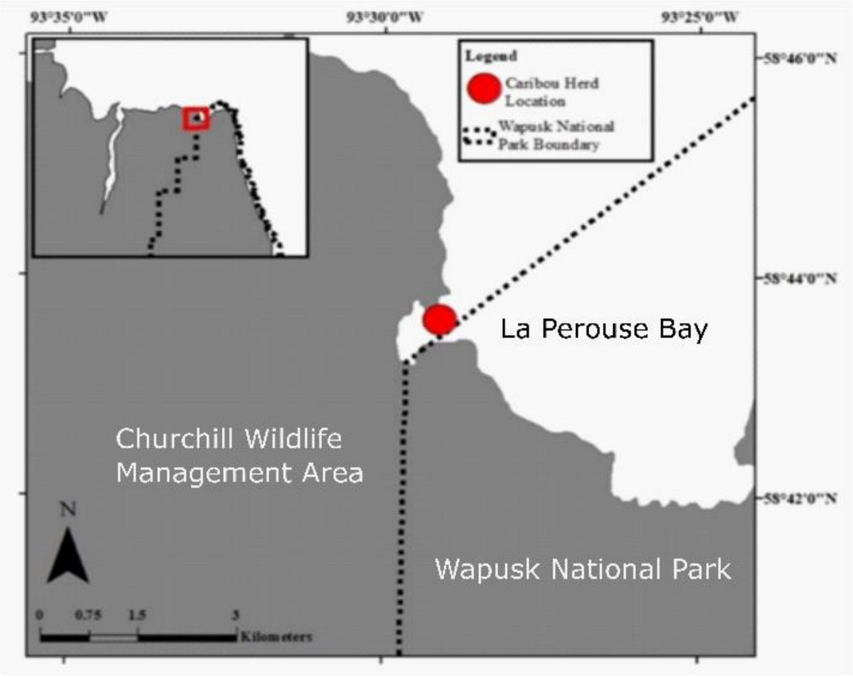

Figure S1.1. Location of study area in the Wapusk National Park, Manitoba, Canada. Map was created using ArcMap v 10.8.1.14362 (https://www.arcgis.com/).

1.2 Drone System and Operation Details

1.2.1 Platform specifications

Flights were conducted with a fixed-wing, rear propelled Trimble UX5 (Figure S2). The Trimble UX5 was black in color with a 100 cm and 2.5 kg weight. It has a cruising speed of 80 km h^{-1} and is powered by a single removable lithium polymer battery (14.8 V, 6000 mAh). The UX5 has an estimated endurance of 50 mins and estimated range ≤ 5 km (see section *2.3 Flight planning and method of operation*). The Trimble UX5 is no longer commercially available from Trimble and has been replaced by the newer Delair UX11 (https://delair.aero/delair-commercial-drones/professional-mapping-drone-delair-ux11/).

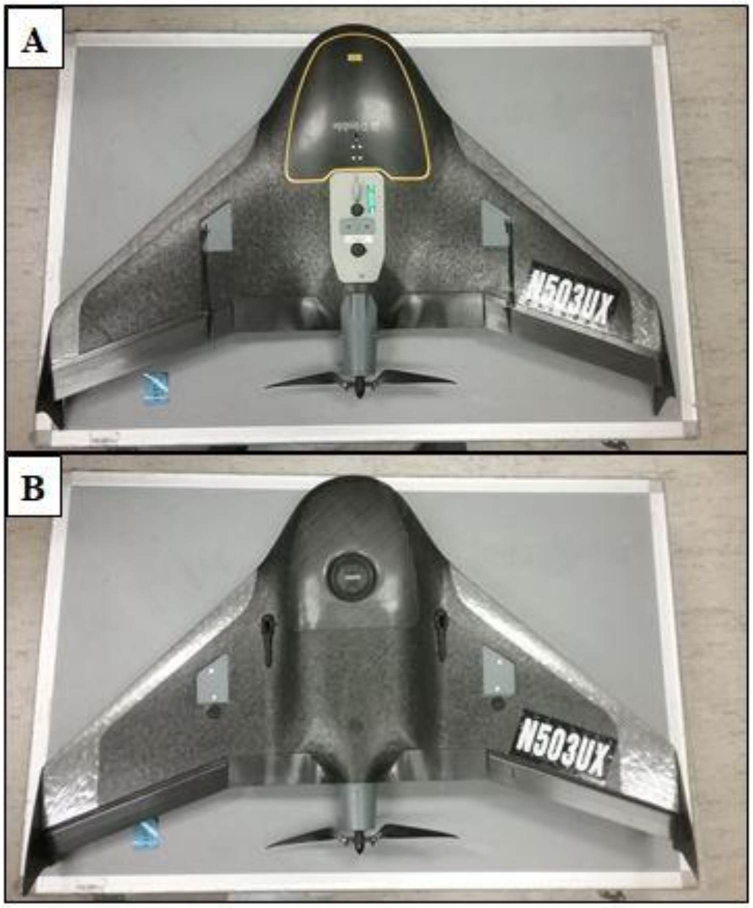

Figure S1.2 An example image of a Trimble UX5 used in the caribou surveys of this study. A) Dorsal view, B) Ventral view with aircraft FAA registration number. Figure was assembled using Affinity Designer v 1.9.1.979 (https://affinity.serif.com/).

1.2.3 Takeoff and retrieval

Prior to takeoff, the pilot-in-command performed a pre-flight checklist for the UX5 and uploaded the pre-programmed flight plan to the drone's onboard navigational system. UX5 takeoffs were initiated using an elastic catapult launcher to ensure a takeoff speed of at least 65 km h^{-1}, which is required to activate the electric motor (Figure S3). For optimal takeoff conditions, the launcher is faced into the prevailing wind direction and at a 45° angle from the ground. No specialized landing or retrieval equipment was used, as the UX5 is designed with a reinforced ventral surface to facilitate "belly landings". Landings require a relatively flat patch of land, approximately 30 × 75 m to accommodate for error or differing environmental conditions during the landing sequence (e.g. GPS error, crosswinds, etc.). All takeoffs during this study occurred within the La Pérouse

Bay research compound, while landings took place adjacent to the compound for extra safety precautions (i.e., to avoid collisions with personnel and buildings).

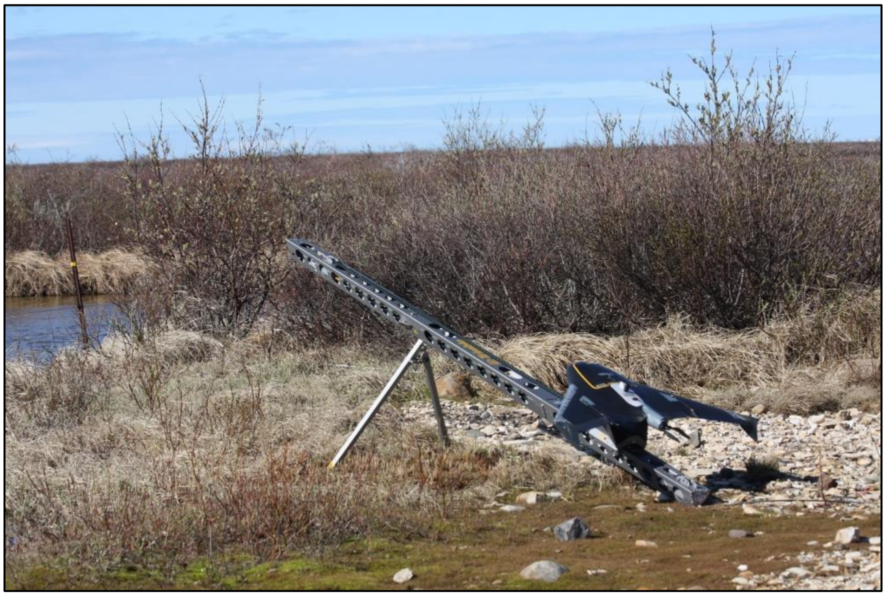

Figure S1.3. The UX5 mounted on its elastic catapult launcher.

1.2.4 Flight planning and method of operation

All flight plans were preprogrammed semiautonomous line transects using Trimble Access Aerial Imaging V2.0.00.40 (Trimble, Sunnyvale, CA). Flight planning involved specifying locations for takeoff and landing, and the desired degree of percent image overlap (which dictated the spacing between adjacent line transects). Direction and orientation of flight lines were determined based on environmental conditions (i.e. wind speed and direction). Programming drone flights in a remote environment required us to download Google Earth background imagery prior to field work (due to a lack of internet connectivity). Real time monitoring of the UX5 position during flight was done using a Trimble Yuma 2 Ground Control Station (GCS), which provided constant feedback on UX5 flight parameters such as: location, distance from GCS, battery level, altitude, and cruise speed. During flight, the UX5 executed flight plans automatically, but the pilot had the ability for safety interventions such as recalling the aircraft to the takeoff/landing site, execute a circular holding pattern, or to abort the flight via immediate landing. In accordance with our Transport Canada Special Flight Operations Certificate (see section *6. Permits, Regulations, Training, and Logistics*), drone operations required the presence of at least two researchers on the ground, a pilot-in-command monitoring the status of the drone using the Yuma GCS, and an observer tasked with observing the UX5 by maintaining visual contact with the drone at all times during flight.

1.3 Payload, Sensor, and Data Collection

1.3.1 Data overview

Surveys of caribou were done by the UX5 collecting still images with 80% vertical and horizontal overlap. Imagery was collected in Red Blue Green format (RBG, 3 visible bands), and saved as jpg files on 316 GB SD cards housed onboard the UX5. Images were geotagged with a latitude and longitude during postprocessing based on a csv file for each camera trigger during the flight and this information was used in the mosaic creation process (see section *5 Data post-processing*). For an example of raw image quality, see Figure S4.

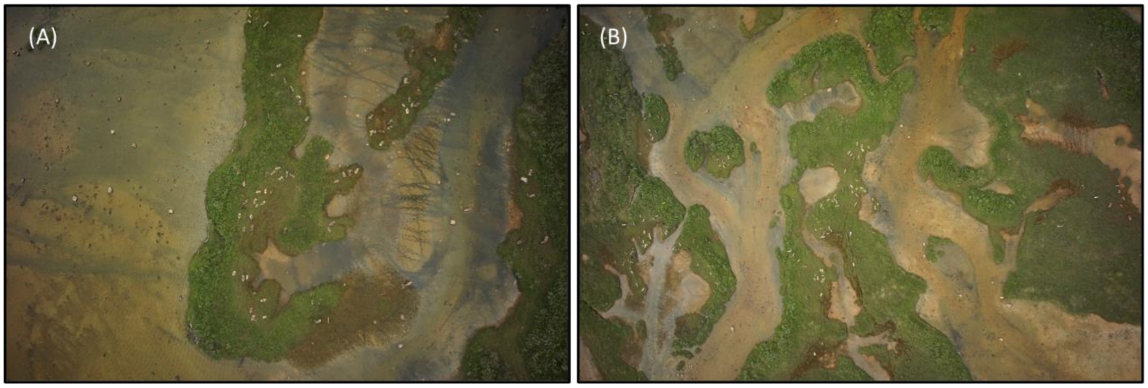

Figure S1.4. Example RGB image taken from the UX5 during a survey of caribou on July 18th 2016. A) 75m flight altitude, B) 120m flight altitude. Figure was assembled using Affinity Designer v 1.9.1.979 (https://affinity.serif.com/).

1.3.2 Payload or sensor description, and data collection methods

The UX5 was equipped with a single payload during surveys, a nadir-oriented Sony NEX-5R 16.1 MP camera (Sony Corporation of America, New York, NY). During flight, images were automatically collected at a rate to achieve the desired degree of image overlap (approximately one per second). Camera settings for all flights were as follows: exposure time 1/4000 s, focal length of 15.5 mm, and automatic white balance. Ground sampling distances of images at 75m and 120m Above Ground Level (AGL) were 2.4 cm and 3.8 cm respectively (see section *4. Field Operation Details*).

1.4 Field Operation Details

Drone surveys in this study were done using the UX5 on 18 July 2016, from 09:08 to 12:41 (seconds not recorded). Four flights were conducted to survey caribou, two at 120m AGL, and two at 75m AGL (Table S1). Average flight duration was 28.5 mins (range 25-32 mins). Weather conditions during flight were cloudy as measured by the pilot-in-command. While operations of the UX5 were limited to within-visual-line-of-sight, fortunately the caribou herd being surveyed remained close to the La Perouse Bay field camp, well within line of sight.

Table S1.1 Summary of drone flights used for surveying caribou along the border of Wapusk National Park, Manitoba, Canada. All flights conducted using the Trimble UX5.

Flight #	Altitude (m AGL)	Launch Time	Land Time	Flight Length (mins)	Pictures
1	120	09:08	09:33	25	697
2	75	10:10	10:42	32	1068
3	120	11:23	11:49	26	700
4	75	12:10	12:41	31	1027
			**Total**	114	3492

1.5 Data Post-Processing

At the conclusion of each flight we downloaded a comma delimited (csv) file with latitude, longitude, altitude, yaw, pitch, and roll for each camera trigger. This information was associated with the respective image in Pix4D (Version 4.x) at the start of the mosaic processing. While not used in this analysis, the post-processing included the product of a digital surface model (DSM) and point cloud. We did not use ground control stations for additional georeferencing since that level of spatial resolution was not needed for the objective of the project. The digital orthomosaic TIFF files created (Figure S5) were then uploaded into the Open UAS Repository – OUR (https://digitalag.org/our/) for ease in viewing and classification of images, as described in the methods section. For full details of image geoprocessing and associated errors, we have included a full Pix4D Quality Report at the end of this document.

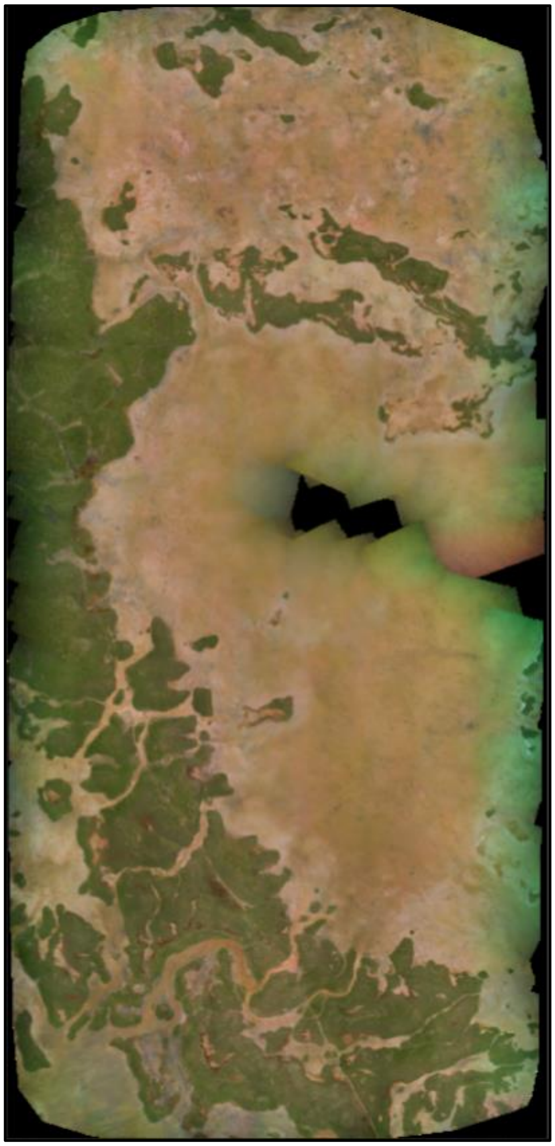

Figure S1.5. Example of orthomosaic produced by Pix4D at 75 m AGL, and uploaded to the Open UAS Repository (https://digitalag.org/our/). Orthomosaic was generated using Pix4D v. 3.1.22 (https://www.pix4d.com/).

1.6 Permits, Regulations, Training and Logistics

Purchase and operation of the UX5 was contingent on attending a training course offered by licenced Trimble partners. Authors AFB and SNE attended a training course on March 18th-20th 2015 in Raleigh, North Carolina. Drone operations for this research were approved by a Transport Canada Special Flight Operations Certificate (File: 5802-11-302, Air Traffic Service: 15-16-00058646, Records, Documents and Information Management System: 11717338), and Wapusk National Park permit WAP-2015-18846. The University of North Dakota Unmanned Aircraft System Research Compliance Committee reviewed human privacy and data management protocols for this project (approved April 10th, 2015). Drone flights of caribou were permitted by the University of North Dakota Institutional Animal Care and Use Committee approvals Protocol #1505-2 for office A3917-01, as well as Canadian Wildlife Service permit 16-MB-SC001 since the primary mission was mapping the common eider colony.

2. Supplementary figures

Figure S2.1. Tiling process consisting in overlapping the 1,000 x 1,000 pixels tiles by 100 pixels, if an individual caribou is shared by Tile 1 and 2, is annotated in Tile 2. Figure was created using Affinity Designer v 1.9.1.979 (https://affinity.serif.com/).

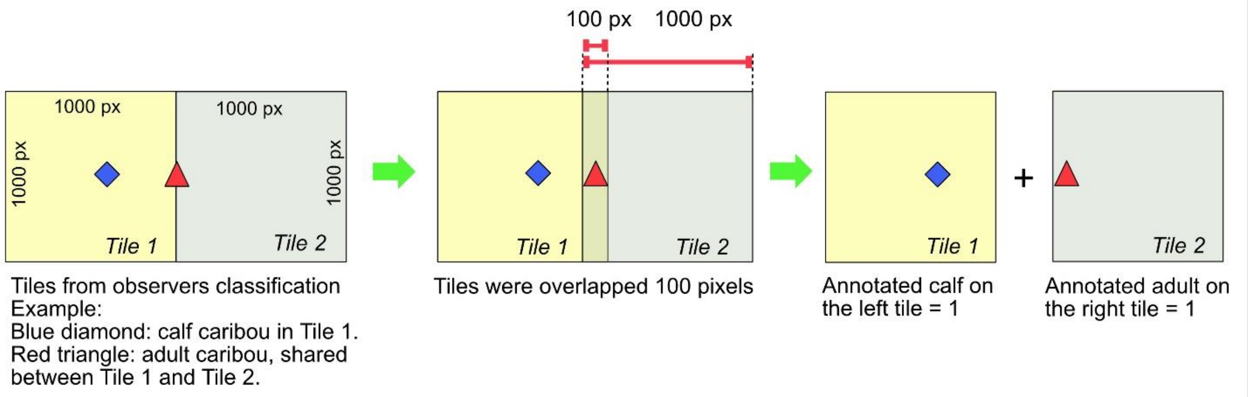

Figure S2.2. Comparison between the proportion of missed caribou of the Faster-RCNN and each of the naïve observers (initials on top of the panels) per mosaic. Figure was created using R v 4.1.3 (R Core Team, 2022).

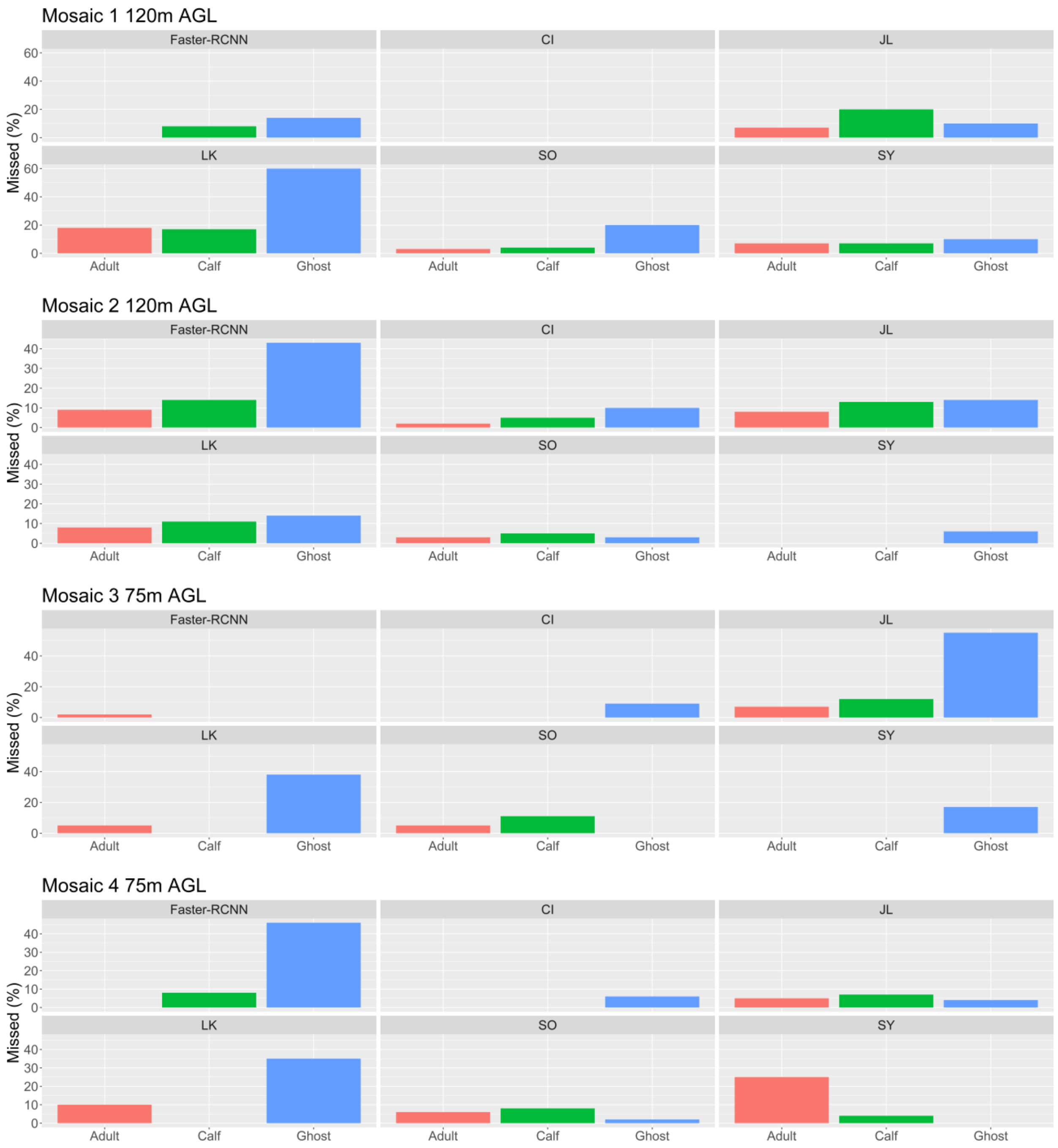

Figure S2.3. Comparison between misclassifications of the Faster-RCNN and each of the naïve observers (initials on top of the panels) per mosaic. Figure was created using R v 4.1.3 (R Core Team, 2022).

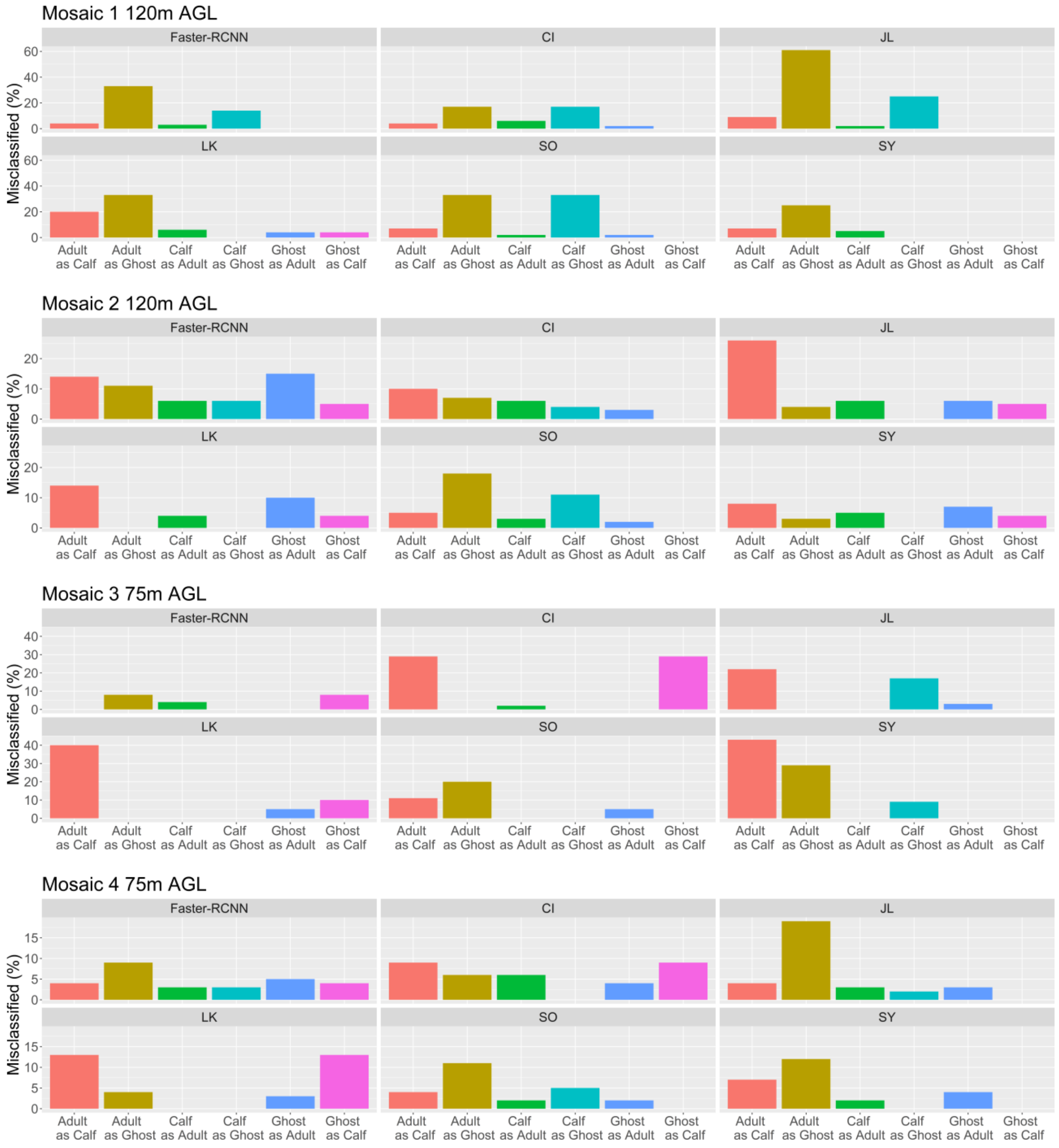

Supplement: Supplementary file 1 — Supplementary Information. [file 41598_2023_28240_MOESM1_ESM.pdf]
